# A Hybrid Method to Enhance Thick and Thin Vessels for Blood Vessel Segmentation

**DOI:** 10.3390/diagnostics11112017

**Published:** 2021-10-30

**Authors:** Sonali Dash, Sahil Verma, Kavita, Md. Sameeruddin Khan, Marcin Wozniak, Jana Shafi, Muhammad Fazal Ijaz

**Affiliations:** 1 Department of Electronics and Communication Engineering, Raghu Institute of Technology (A), Visakhapatnam 531162, Andhra Pradesh, India; sonali.isan@gmail.com; 2 Department of Computer Science and Engineering, Chandigarh University, Mohali 140413, India; sahilverma@ieee.org (S.V.); kavita@ieee.org (K.); ed.cse@cumail.in (M.S.K.); 3 Bio and Health Informatics Research Lab, Chandigarh University, Mohali 140413, India; 4 Machine Learning and Data Science Research Lab, Chandigarh University, Mohali 140413, India; 5 Vision and Learning Research Lab, Chandigarh University, Mohali 140413, India; 6 Faculty of Applied Mathematics, Silesian University of Technology, 44-100 Gliwice, Poland; 7 Department of Computer Science, College of Arts and Science, Prince Sattam bin Abdul Aziz University, Wadi Ad-Dwasir 11991, Saudi Arabia; j.jana@psau.edu.sa; 8 Department of Intelligent Mechatronics Engineering, Sejong University, Seoul 05006, Korea

**Keywords:** blood vessel segmentation, curvelet transform, Jerman filter, mean-C thresholding

## Abstract

Retinal blood vessels have been presented to contribute confirmation with regard to tortuosity, branching angles, or change in diameter as a result of ophthalmic disease. Although many enhancement filters are extensively utilized, the Jerman filter responds quite effectively at vessels, edges, and bifurcations and improves the visualization of structures. In contrast, curvelet transform is specifically designed to associate scale with orientation and can be used to recover from noisy data by curvelet shrinkage. This paper describes a method to improve the performance of curvelet transform further. A distinctive fusion of curvelet transform and the Jerman filter is presented for retinal blood vessel segmentation. Mean-C thresholding is employed for the segmentation purpose. The suggested method achieves average accuracies of 0.9600 and 0.9559 for DRIVE and CHASE_DB1, respectively. Simulation results establish a better performance and faster implementation of the suggested scheme in comparison with similar approaches seen in the literature.

## 1. Introduction

Evaluation of the physical features of the retinal vascular structure can create understanding of the pathological transformation generated by ocular diseases. The illustration of the retinal vasculature is significant for analysis, treatment, viewing, assessment, and the clinical study of ophthalmic diseases that include retinal artery occlusion, diabetic retinopathy, hypertension, and choroidal neovascularization [1,2]. Blood vessels are dominating and mainly steady structures, which appear in the retina that is detected directly in vivo. The efficacy of cure for ophthalmologic disorders is dependent on the prompt recognition of alteration in retinal pathology. The manual labelling of retinal blood vessels is a tedious procedure that requires training and skill. Computerized segmentation offers reliability and accuracy and decreases the consumption of time by a physician or a skilled technician for hand mapping. Thus, an automatic definitive approach of vessel segmentation would be beneficial for the prior recognition and characterization of morphological alterations in the retinal vasculature. Generally, the feature representation and extraction in retinal images is a difficult assignment. The foremost complications are the lighting changes, insufficient contrast, noise effect, and anatomic changeability dependent on the individual patient.

Many filters are suggested for the enhancement of retinal blood vessels. Jerman et al. have recommended an improved multiscale vesselness filter, based on the ratio of multiscale Hessian eigenvalues, that produces uniform and stable acknowledgement in all vascular structures and correctly improves the border between the vascular structure and the background, later known as the Jerman filter [3,4]. They have assessed the proposed enhancement filter on High-Resolution Fundus (HRF) image database. However, their suggested method is only limited to the enhancement of retinal vasculature more uniformly. Frangi et al. have suggested a filtering approach with multiscale for vessel enhancement called the Frangi filter [5].

Among the human organs, the retina is the only place in which blood vessels can be captured straight noninvasively in vivo. In the automatic retinal disease recognition, blood vessels play a vital role, as they comprise the essential topic of screening systems. Precise segmentation and analysis of blood vessel length, thickness, and orientation can simplify the assessment of retinopathy of prematurity and recognition of arteriolar narrowing assessment of vessel diameter for the detection of aliments such as hypertension, diabetes, and arteriosclerosis, etc. [6].

On the other hand, many researchers have shown the importance of mutiresolution analysis, especially applicable for the enhancement of retinal blood vessels [7,8]. Among the multiresolution analysis, curvelet transform is one of the important techniques for enhancement of blood vessels. Few efforts have been introduced for enhancing the performances of curvelet transform by extending it in various ways for the segmentation of retinal images. Esmaeili et al. have offered a new technique for enhancing the retinal blood vessels utilizing curvelet transform [9]. In 2011, Miri and Mahloojifar utilized curvelet transform for the detection of the retinal image edges effectively through multistructure morphology operators [10]. In 2016, Aghamohamadian-Sharba et al. (2015) utilized curvelet transform to automatically grade the retinal blood vessel tortuosity [11]. In 2014, Kar et al. combined curvelet transform with matched filter and conditional fuzzy entropy for extraction of blood vessels [12]. In 2016, the same authors suggested another method for extraction of blood vessels by combining curvelet transform with matched filter and kernel fuzzy c-means [13]. Some of the significance associated with supervised and unsupervised works for retinal vessel segmentation that are available are discussed below.

Numerous principles and approaches for retinal vessel segmentation have been described in the literature. Fraz et al. have given a detailed report for the various approaches available for the retinal vessel segmentation [14]. Detection of retinal blood vessel segmentation can be designated into procedures on the basis of pattern recognition, vessel tracking, match filtering, multiscale analysis, morphological processing, and model-based algorithms. Furthermore, the pattern recognition techniques can be distributed into two classes: supervised approaches and unsupervised approaches. Supervised approaches use ground truth data for the classification of vessels that consider features of the blood vessels. These approaches comprise principal component analysis [15], neural networks [16], k nearest neighbour classifiers [17], and support vector machine (SVM) [18]. Some of the unsupervised approaches include matched filtering along with specially weighted fuzzy c-means clustering [19], radius-based clustering algorithm [20], and maximum likelihood estimation of vessel parameters [21].

Generally, the green channel of the image is taken into consideration in most of the vessel segmentation methods because of low level of noise and high level of contrast. Soares et al. have recommended an approach that classified pixels as vessel or nonvessel by utilizing supervised classification [22]. Lupascu et al. have utilized AdaBoost for the construction of a classifier [23]. Chaudhuri et al. have proposed methods based on matched filtering that convolves with a 2D template and designed to form the characteristics of the vasculature [24]. Kovacs and Hajdu have also suggested an approach considering matching of template and contour reconstruction [25]. Annunziata et al. have suggested an approach in which the presences of exudates in retinal images are reported [26]. Zamperini et al. have classified vessels considering the contrast, size, position, and colour by investigating the nearby pixels of background [27]. Relan et al. have utilized Gaussian Mixture Model with a hope to maximize clustering for the classification of vessels [28]. Dashtbozorg et al. have suggested a new approach to classify considering the geometry of vessels [29]. Estrada et al. have also taken graph theoretical method into consideration by extending a global likelihood model [30]. Relan et al. have employed the least square-support vector machine approach for the classification of veins on four-color features [31]. Vascular tortuosity measurement is important for the diagnosis of diabetes and some diseases related to central nervous system. Hart et al. have suggested a tortuosity measurement and classification of vessel segmentation and networks and also summarized the previous works [32]. Grisan et al. have recommended a tortuosity density measure to deal with the vessel segmentation of various lengths by adding each local turn [33]. Lotmar et al. have originated the first approach of the first kind that is extensively used [34]. Poletti et al. have suggested combined approaches for image-level tortuosity estimation [35]. Angle of variation for tortuosity valuation for the diagnosis of retinopathy is considered by Oloumi et al. [36]. Five approaches based on different principles are compared by Lisowska et al. [37]. Perez Rovira et al. have suggested a complete system for vessel analysis, which utilized the tortuosity measure by Trucco et al. [38,39]. Azzopardi et al. have recommended vessel extraction by using the B-COSFIRE (Combination of Shifted Filter Responses) technique that provides rotation invariance effectively by shifting operations [40]. Mapayi et al. have suggested of blood vessel segmentation by using GLCM (Gray Level Co-occurrence Matix) energy information through an adaptive thresholding technique [41]. Zhao et al. have proposed an infinite perimeter active contour model through combining intensity information and local phase-based enhancement map for vessel extraction [42]. Zhang et al. have suggested a new approach of segmenting the blood vessel in two ways: one is using LID (left invariant rotating derivative), and the other one is LAD (locally adaptive derivative) frame. The results of multiscale filtering through LAD or LID give enhanced images for vessel extraction [43].

Tan et al. have proposed an automated approach for extraction of retinal vasculature in which they have filtered the retinal images via a bank of Gabor kernels. The outputs are combined to form a maximal image that is thinned to get a network of one-pixel lines, examined and clipped to locate forks and from branches. Lastly, for finding out salient points, the algorithm known as Ramer–Douglas–Peucker is employed [44]. Farokhain et al. have designed new sets of Gabor filters applying imperialism competitive algorithm for blood vessel segmentation [45]. Orlando et al. have recommended vessel segmentation by conditional random field model [46]. Rodrigues and Marengoni have employed a graph-based technique utilizing Dijkstra’s shortest path algorithm and a statistic t distribution to extract the blood vessel [47]. Jiang et al. have recommended a pretrained fully convolutional network through transfer learning for the segmentation of blood vessels [48]. Khomri et al. have proposed a vessel segment method using the Elite-guided Multi-Objective Artificial Bee Colony (EMOABC) algorithm [49].

Memari et al. have suggested fuzzy c-means clustering integrated with level sets for blood vessel segmentation. They have employed contrast limited adaptive histogram equalisation, mathematical morphology integrated with a matched filter, the Gabor filter, and the Frangi filter to reduce the noise and enhance the retinal images [50]. Sundaram et al. have integrated few existing techniques such as morphological operations, bottom hat transform, multiscale vessel enhancement (MSVE) algorithm, and image fusion for retinal vessel segmentation [51]. Recently, Dash and Senapati have introduced an extension of Discrete wavelet transform (DWT) by combining it with the Coye filter [52]. Dash et al. have enhanced the image by using homomorphic filter-based enhancement to enhance the features. Afterwards, vessels are extracted by utilizing K-mean clustering method [53]. Likewise, there are many other suggested works are available in the literature for the improvement and detection health diseases utilizing machine learning [54,55,56,57,58] and deep learning [59,60,61,62,63,64].

Glaucoma is the second main basis of irreparable blindness worldwide. The disease progression of glaucoma can be controlled by early detection. Glaucoma leads to deterioration of optic nerves. The ratio of the optic cup to the optic disc, also known as the cup-to-disc ratio (CDR), is one of the important and standard measures to identify glaucoma. Trained ophthalmologists determine the CDR value manually, which restricts en masse screening for early detection of glaucoma. The exact value of CDR is difficult to determine if the optic cup and optic disc are not thoroughly distinct [65]. Therefore, an optic disc improvement technique is highly recommended. The suggested work can improve the quality of optic disc and optic cup for glaucoma identification.

Even though, recently, deep learning has been successfully implemented for retinal blood vessel segmentation, a lot can still be done to ameliorate the traditional unsupervised approaches further. Wang et al. have recommended Context Spatial U-Net for blood vessel segmentation [66]. Generally, designing a method of enhancing the contrast that generates a visual-artifact-free output is impracticable. Selection of a particular enhancement system is challenging due to the absence of reliable parameters for the evaluation of the quality of the output image. 

Even though most of these approaches have generated prominent results, they so often present various complications because of noise and the imprecise nature of the retinal vessel images. In such circumstances, numerous critical challenges still exist that must be addressed, for instance, occurrence of false positives, poor connectivity in retinal vessels, accuracy under noisy condition, and many more. Despite few efforts having been taken for the enhancement of retinal vessel using curvelet transform, the improvement of the performance of curvelet transform can still be a challenging task. 

Curvelet has the advantage of modifying the curvelet coefficients; it has the ability to enhance the edges more precisely. The multistructure elements approach has the property of directionality feature that makes it an effective tool in edge detection. The curvelet decomposition has the benefit of denoising as well as highlighting the edges and vessel curvature. The Jerman filter has the advantage of producing uniform and stable effect in all vascular structures and correctly improves the border in between the vascular structure and the background. 

In this paper, a new attempt has been made by considering the advantages of both Jerman filter and curvelet transform for retinal vessels enhancement and mean-C thresholding for segmentation. The suggested approach integrates two different techniques, Jerman filter and curvelet transform, to improve the performance of the curvelet transform.

The paper is organized as follows: Section 2 discusses the methodology in detail. Results and comparisons are given and discussed in Section 3. In Section 4, the conclusions are drawn.

## 2. Materials and Methods

**Figure 1:**
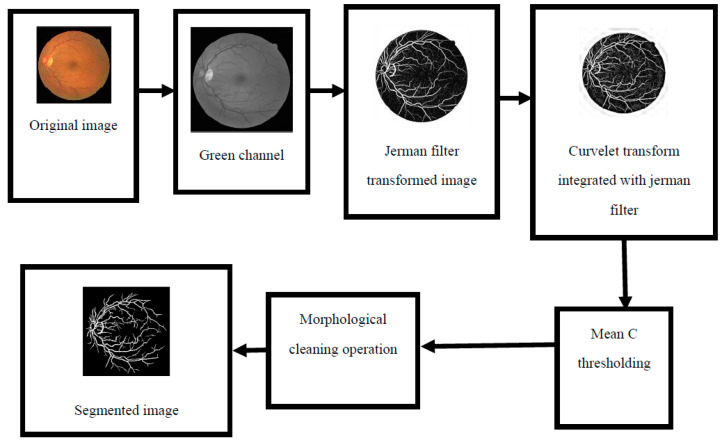
Schematic outline of the suggested methodology.

The suggested execution of the retinal blood vessel extraction system is enlightened by curvelet transform integrated with the Jerman filter [4]. The main purpose of combining the two techniques is to improve the traditional curvelet transform performance further for the enhancement of retinal blood vessels. Figure 1 shows the functional block diagram of the suggested segmentation scheme, and there are three stages of the approach that are comprehensively described below.

### 2.1. Preprocessing

Due to the difficulties in capturing pictures of retinal images through pupil, the blood vessels have unbalanced illumination. Because of the low contrast, the vessels in the dark areas are difficult to discern. Therefore, the whole preprocessing stages are critical in order to extract as many fine vessels as possible. Accordingly, the overall image quality requires improvement through the preprocessing steps. Thus, the suggested method is a unique combination of a Jerman filter with curvelet transform to improve the performance measures of blood vessels.

**Figure 2:**
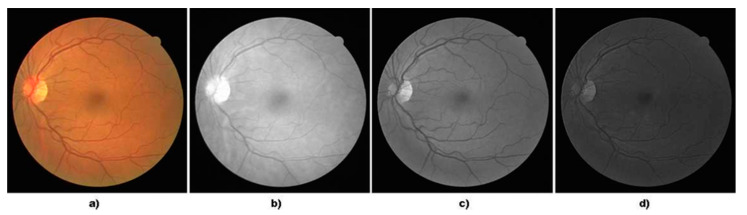
(**a**) Colour Image, (**b**) Red Channel, (**c**) Green Channel, (**d**) Blue Channel.

It can be inferred from Figure 2 that the vessels are better distinguishable in the green channel in comparison to the blue and red one. Hence, the green channel is considered in the entire process of vessel extraction.

#### 2.1.1. Enhancement of Vasculature Jerman Filter

Although various enhancement filters are extensively utilized, the responses of the filters are not uniform in between vessels of distinct radii. A close-to-uniform response is achieved for the entire vascular structure by initially considering the filter that uses the ratio of multiscale Hessian eigenvalues. 

To handle with the deviations of intensity and outline of the targeted structures, noise, etc., the indicator function is approximated by smooth enhancement functions. Through maximization of a specified enhancement function, a multiscale filter response F(x) is then achieved, at each point x, over a span of scale s, as given below:

(1) $$F\left(x\right)=\sup\left\{\nu \left[\mathrm{eig}\left(x,s\right)\right]:s_{\min}\leq s\leq s_{\max}\right\}$$

where H(x,s) is the hessian of F(x) at x and scale s.

The vasculature mostly contains straight vessels and rounded structures such as bending vessels and bifurcations and vascular pathologies, for example, aneurysms. The elongated tube type structures, for instance, vessels, can be enhanced by considering the eigenvalue λ_m_ of H (x, s), m = 1, 2, …E that can be calculated fast for 2 × 2 Hessian matrices in 2D images with the analytic approach. The elongated structures are specified by |λ_2_|>>|λ_1_| for 2D images (E = 2) in which λ_2_ is a bright (dark) structure on a dark (bright) background. For differently shaped structures, the eigenvalue relationships can be achieved in the same way. 

Frangi et al. (1998) suggested a function in which a factor is used to suppress the rounded structures, as described below [5]:

$\exp\left(-\frac{{ℜ}_{B}^{2}}{2\beta^{2}}\right)$, with ${ℜ}_{B}=\left|\lambda_{1}\right|/{\surd \lambda }_{2}\lambda_{3}$ and β=constant.

Jerman et al. (2016) removed the factor and reduced Frangi’s function as:

(2) $$\nu_{F}=\left(1-\exp\left(-\frac{{ℜ}_{A}^{2}}{2\alpha^{2}}\right)\right)\left(1-\exp\left(-\frac{S_{3}^{2}}{2K^{2}}\right)\right)$$

where $S_{3}=\sqrt{\lambda_{1}^{2}+\lambda_{2}^{2}+\lambda_{3}^{2}}$ is the second order measure of image structure that is designated as $S_{D}=\sqrt{\underset{i\leq D}{\sum }\lambda_{i}^{2}}$, where D indicates dimension, and ${ℜ}_{A}=\frac{\lambda_{2}}{\lambda_{3}}$ discriminates between tubular and planar structures. Parameters α and K control the sensitivity of the measures ${ℜ}_{A}$ and S, respectively. In 2D, the corresponding transformed Frangi’s enhancement function comprises only the second factor of Function (1), which is given as:

(3) $$\nu_{F}=\left(1-\exp\left(\frac{S_{2}^{2}}{2K^{2}}\right)\right)$$

where $S_{2}=\sqrt{\lambda_{1}^{2}+\lambda_{2}^{2}}$.

The original Sato’s enhancement function consists the factor ${\left(1+\frac{\lambda_{1}}{\left|\lambda_{2}\right|}\right)}^{\delta }$, δ ≥ 0 for the suppression of rounded structures (Sato et al. 2000) [67]. By eliminating this aspect, Jerman et al. (2016) derived the equation as [4]:

(4) $$\nu_{S}=\left|\lambda_{3}\right|{\left(\frac{\lambda_{2}}{\lambda_{3}}\right)}^{\gamma }$$

where γ controls the sensitivity.

Li et al. (2003) suggested an alike enhancement function that is without the suppression of spherical structures, stated as [7]:

(5) $$\nu_{S}=\frac{\lambda_{2}^{2}}{\left|\lambda_{3}\right|}$$

that can be factored into $\left|\lambda_{2}\right|\times \lambda_{2}{/\lambda }_{3}$. As reported by to Li et al. (2003), the first factor denotes the magnitude and the second likelihood of an elongated structure.

The terminologies of the entire abovementioned enhancement functions are, by some means, proportional to the magnitude or squared magnitude of $\lambda_{2}$ or $\lambda_{3}$. By utilizing $e^{x}\approx 1+x$ to approximate the second order factor in Function (1), Jerman et al. (2016) derived the following [4].

(6) $$\left(1-\exp\left(-\frac{S^{2}}{2K^{2}}\right)\right)\frac{1}{2K^{2}}\left(\lambda_{1}^{2}{+\lambda }_{2}^{2}{+\lambda }_{3}^{2}\right)$$

That explains Frangi’s Function (1) is proportional to the squared magnitude of $\lambda_{2}$ and $\lambda_{3}$.

The dependency of the enhancement functions on the magnitude of $\lambda_{2}$ or $\lambda_{3}$ is executed mostly to suppress the noise in image regions with low and uniform intensities in which all eigenvalues have low and alike magnitudes.

For regularizing the value of $\lambda_{3}$ at each scale p, the following formulation is considered:

(7) $$\lambda_{m}\left(p\right)=\{\begin{array}{c} \lambda_{3}\;\mathrm{if}\;\lambda_{3}>{\tau \max}_{x}\lambda_{3}\left(x,\;p\right)\\ {\tau \;\max}_{x}\lambda_{3}(x,\;p)\;\mathrm{if}\;0\;<\;\lambda_{3}\;\leq \;{\tau \max}_{x}\lambda_{3}\left(x,\;p\right)\\ 0\;\mathrm{otherwise}\; \end{array}$$

where τ is the cut-off threshold between 0 and 1.

By this eigenvalue regularization mentioned above, the enhancement function can be described individually of the relative brightness of the structures of importance as follows:

(8) $$\upsilon =\lambda_{2}^{2}\lambda_{m}{\left[\frac{3}{{2\lambda }_{2}{+\lambda }_{m}}\right]}^{3}$$

Accordingly, Jerman et al. (2016) derived the enhanced filter function as [4]:

(9) υa={       0      if λ2 ≤ 0 ∨λm ≤ 0       1      if λ2 ≥λm2 > 0 λ22(λm−λ2)[32λ2+λm]3     otherwise     

**Figure 3:**
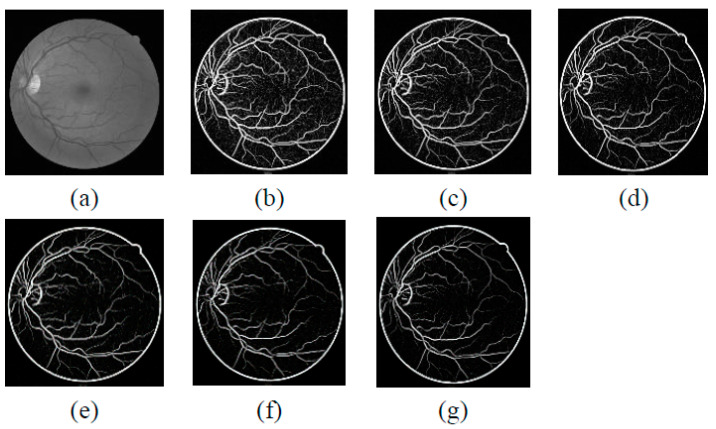
Comparison of green channel- and Jerman-filtered images with different τ values. (**a**) Green channel, (**b**) τ = 0.5, (**c**) τ = 0.6, (**d**) τ = 0.7, (**e**) τ = 0.8, (**f**) τ = 0.9, (**g**) τ = 1.

**Figure 4:**
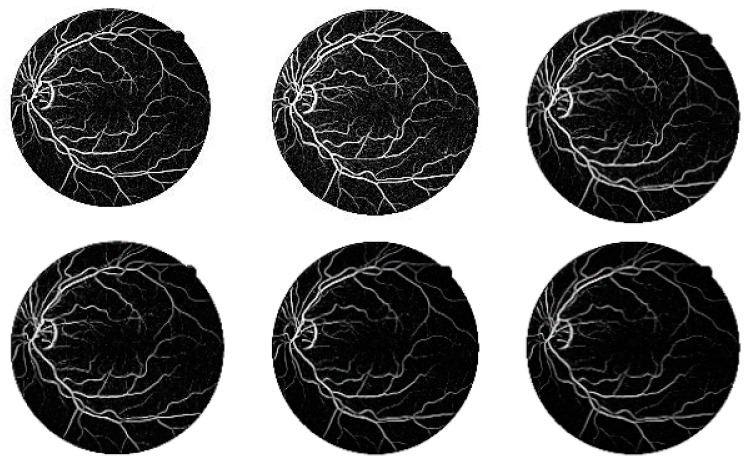
Retinal images obtained from a Jerman filter after the disks are removed for different τ values.

The suggested enhancement function is considering a ratio of eigenvalues with range response values from 0 to 1. Figure 3a presents the green channel, and Figure 3b,g shows the images obtained by applying the Jerman filter on the green channel of the blood vessel. Figure 4 displays the filtered images after the disk is removed. 

#### 2.1.2. Enhancement of Vasculature by Curvelet Transform

The Jerman-filtered transformed images are further processed through curvelet transform. The purpose of choosing curvelet transform is explained below.

In the curvelet transform, the curvelets are designed to pick up curves utilizing only a small number of coefficients. Therefore, the curve discontinuities are managed finely with curvelets. Main advantages of curvelet transform are its sensitivity in the direction of directional edges and contours and its ability to signify them by few sparse nonzero coefficients. Thus, in comparison to wavelet transform, curvelet transform can proficiently illustrate the edges and curves with a smaller number of coefficients. Furthermore, curvelet transforms are utilized to enhance the contrast of an image by highlighting its edges in several scales and directions. 

The details of mathematical formulations are discussed as follows.

Donoho and Ducan (2000) suggested curvelet transform that is derived from ridgelet transform [8]. The curvelet transform is appropriate for objects that are smooth away from discontinuities across curves. Curvelet transform handles curve discontinuities in a fine manner because it is designed to handle curves utilizing only a small number of coefficients. The multiwavelet transformation offers better spatial and spectral localization of an image when compared with other multiscale representations. However, here, the curvelet via wrapping is implemented, as it is faster and has less computational complexity. In this method, the Fourier plane is divided into a number of concentric circles referred to as scale; each of these concentric circles is again divided into a number of angular divisions referred to as the orientation. This combination of the scale and the angular division is known as parabolic wedges. As these radial wedges capture the structural activity in the frequency domain, high anisotropy and directional sensitivity are the inherent characteristics of the curvelet transform. Next, to find out the curvelet coefficients, inverse FFT is taken on each scale and angle. The curvelet transform consists of four stages and is implemented as given below.

Initially in the subband decomposition, the image is first decomposed into log_2_N (N is the size of the image) wavelet subbands, and then curvelet subbands are formed by forming partial reconstruction from these wavelet subbands at various levels. The subband decompositions are denoted as:

$$f\to \left(P_{0}f,\Delta_{1}f,\Delta_{2}f,\ldots .\right)$$

where P_0_→ lowpass filter, Δ→ bandpass (highpass) filters. 

The image is distributed into resolution layers ${\;P}_{0}$. All layers include the particulars of various frequencies. In the next step of smooth portioning, every subband is smoothly windowed into ‘squares’ of a suitable measure. A grid of dyadic squares is described as:

(10) $$I_{\left(s,{\;k}_{1},k_{2}\right)}=\left[\frac{k_{1}}{2^{s}},\;\frac{k_{1}+1}{2^{s}}\right]\times \left[\frac{k_{2}}{2^{s}},\;\frac{k_{2}+1}{2^{s}}\right]\in I_{s}$$

Let p be a smooth windowing function. For every square, P_I_ is a displacement of P localized close to I. The multiplication of Δ_s_f with P_I_ yields a smooth dissection of the function into ‘squares’.

(11) $$h_{I}=P_{I}\Delta_{s}f$$

This stage follows the windowing partition of the subbands isolated in the former step of the algorithm.

(12) $$\Delta_{s}f\to \to P_{I}\Delta_{s}f\;I\in I_{s}$$

In the next step of renormalization, every resultant square is renormalized to unit scale. For a dyadic square I, let the following define an operator that transports and renormalizes f so that the part of the input supported near I becomes the part of output supported near the unit square.

(13) $$T_{I}f\left(x_{1}x_{2}\right)=2^{s}f\left(2^{s}x_{1}-k_{1},2^{s}x_{2}-k_{2}\;\right)$$

In this step, each square resulting in the previous step is renormalized to unit scale.

(14) $$g_{I}=T_{I}^{-1}h_{I}$$

where $T_{I}$ is the operator, and $T_{I}^{-1}$ is the inverse opearator.

Finally, inverse curvelet transform is applied to achieve the curvelet enhanced image.

The digital curvelet transform applied on a 2D image f(x,y), such that 0 < x ≤ M and 0< y ≤ N, gives a set of curvelet coefficients $C\left(s,\theta ,k_{1},k_{2}\right)$ as follows.

(15) $$C\left(s,\;\theta ,{\;k}_{1}k_{2}\right)=\overset{0<x\leq M}{\underset{0<y\leq N}{\sum }}f\left(x,y\right)\varphi_{s,\theta ,k_{1}k_{2}}\left(x,\;y\right)$$

**Figure 5:**
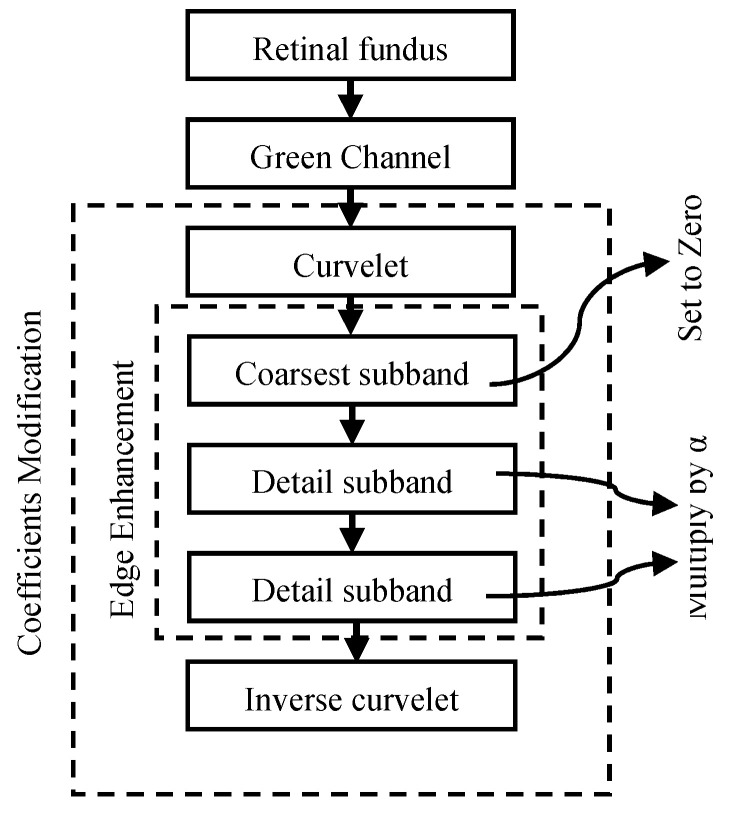
General steps of curvelet transform.

**Figure 6:**
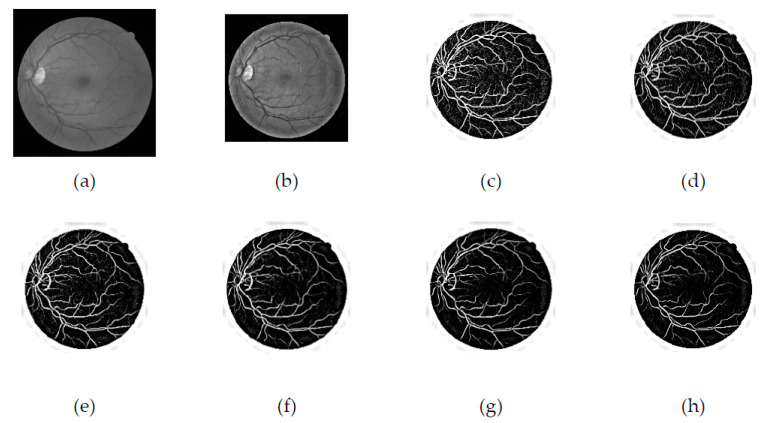
Effect of curvelet transform on green channel- and Jerman-filtered images (**a**) Green channel, (**b**) Curvelet transformed image of green channel, (**c**–**h**) Jerman filter integrated with curvelet transform for different values of τ.

Here, ‘s’ is the scale or no of decomposition level, ‘θ’ is the orientation, ‘k_1_′ and ‘k_2_′ are spatial location of curvelet, and φ and ‘f(x,y)’ are the image in spatial domain. As the decomposition level increases, the curvelets become thinner and sharper. The schematic diagram of the general steps of the curvelet transform is given in Figure 5. Furthermore, the enhanced images obtained from curvelet transform are presented in Figure 6. Figure 6b represents the curvelet transformed image on the green channel. Figure 6c–h presents the enhanced curvelet transform retinal images through the Jerman filter.

### 2.2. Mean-C Thresholding

In this research, mean-C thresholding method is considered in which a threshold is computed for every pixel in the image based on some local statistics such as mean and median. The threshold is updated every time. The core benefit of this approach is that it can be applied to unevenly illuminated images. The steps of the mean-C thresholding are described as follows. 

The novel mean-C thresholding proceeds in two steps—background elimination and vessel segmentation. For background elimination, a mean image is first generated by convolving the enhanced image with a mean filter of window size ‘W’. This average filter smooths the background for the poorly illuminated image. This mean filtered image is subtracted from the enhanced image to produce a difference image. Now with an appropriate threshold value ‘C’, the image is binarised. The values of the parameters ‘W’ and ‘C’ are chosen empirically [58].

- Initially, the mean filter with window size N × N is chosen.
- The transformed image achieved through all the processes is convolved with the mean.
- By taking the difference of the convolved image and the transformed image, a new difference image is obtained.
- The difference image is thresholded with the constant value C. Experimentally, the value of C is fixed as 0.039.
- The complement of thresholded image is computed.

### 2.3. Summary of the Proposed Method

The summary of the contribution of the suggested method is given below.

**Step 1:** Extract the green channel from the fundus image.

**Step 2:** Apply the Jerman filter with different τ value that is the cut-off threshold between 0 and 1. In the experiment, τ value varies from 0.5 to 1.

**Step 3:** Afterwards, Jerman-filter-enhanced images are applied on curvelet transform to improve the curvelet transform. 

**Step 4:** Curvelet decomposition of level ‘s’ = 5 and orientation θ = 16 are applied to each channel individually (as described in Equation (15)). It gives a set of curvelet coefficients for each level of decomposition (i.e. for s = 1, 2.., 5). As the level of decomposition increases, the curvelet becomes thinner and finer, and hence, the s=1 corresponds to the core’s region, and s=5 corresponds to the finest or high-frequency region. The increase in θ value increases the no of coefficient significantly. Hence, the no of decomposition level and the orientation are chosen very precisely to achieve the best representation of the curves and singularities with minimum complexity.

**Step 5:** Then the image enhancement is performed by modifying the curvelet coefficients in such a way that the high-frequency curvatures and edges are emphasized, and the low-frequency cores regions are deemphasized. This coefficient modification scheme is unique and specific to the problem. Here, all the coefficients for s = 1 are set to zero, and the coefficients for all other values of s are multiplied by ‘α’. The value of ‘α’ is fixed at 1.2.

**Step 6:** Apply Inverse Curvelet transform on the modified set of curvelet coefficient to produce the enhanced image. As the coefficient modification set all core levels to zero and fine level coefficients are enhanced, in the reconstructed image, the background appears darker, and the curvatures and edges are highlighted. Various authors have suggested several strategies for coefficient modification; however, the suggested modification is very simple and straightforward and gives efficient enhancement.

**Step 7:** Mean C thresholding is applied on the curvelet-enhanced images to produce the vessel network.

**Step 8:** Mean C thresholding result contains some small, disconnected, vessel-like structures. These may be due to the noise. Thus, a postsegmentation fine tuning is performed by morphological opening operation, which successfully removes the artefacts.

## 3. Results

The performance of the recommended approach is analysed and compared with the other approaches by implementing it on publicly available DRIVE and CHASE_DB1 databases. The DRIVE database images are divided into two sets—training data set and testing data set. Each data set consists of 20 retinal colour images, corresponding mask, and two sets of corresponding manually segmented results. The manual segmented result given by the first ophthalmologist is treated as the ground truth image. The training data set is usually used in supervised methods to train the network. As the suggested scheme is an unsupervised method, we have considered only the test data set.

The CHASE_DB1 (Child Heart and Health Study in England) dataset contains of child retinal images of both the eyes. The images are taken at 30° field of view with resolution of 960 × 999 pixels by hand-held NM-200-D fundus camera. The images consist of uneven illumination at the background and poor contrast blood vessels. Segmentation results of the first of the two observers are deployed as the ground truth. 

To analyse and quantify the method’s efficiency the segmented result is compared with the ground truth and several performance measures like sensitivity, accuracy, and specificity are computed, as per the equation defined below. The accuracy is defined as the ability of algorithm to differentiate the vessel and nonvessel pixels correctly. To estimate the accuracy, one has to calculate the proportion of true positive and true negative in all evaluated cases. The accuracy displays conventionality of the segmentation result. Mathematically, it is defined as:

(16) $$\mathrm{Accuracy}=\frac{\mathrm{TP}+\mathrm{TN}}{\mathrm{TP}+\mathrm{FN}+\mathrm{TN}+\mathrm{FP}}$$

Sensitivity quantifies the techniques of capability to identify the correct vessel pixel. Mathematically, it is stated as:

(17) $$\mathrm{Sensitivity}=\frac{\mathrm{TP}}{\mathrm{TP}+\mathrm{FN}}$$

While specificity is a measure of the capability to identify the background pixels.

(18) $$\mathrm{Specificity}=\frac{\mathrm{TN}}{\mathrm{TN}+\mathrm{FP}}$$

(19) $$\mathrm{Precision}=\frac{\mathrm{TP}}{\mathrm{TP}+\mathrm{FP}}$$

where TP, TN, FP, and FN are defined as follows:

True positive (TP) = counts pixel is accurately recognized as a vessel.

False positive (FP) = counts pixel is inaccurately recognized as vessel.

True negative (TN) = counts pixel is accurately recognized as background.

False negative (FN) = counts pixel is inaccurately recognized as background.

The first experiment is carried out for the original curvelet transform on both DRIVE and CHASE_DB1 databases. Table 1 shows the performance measures of the retinal blood vessel segmentation utilizing original curvelet transform with c-mean thresholding on DRIVE database. The average sensitivity, specificity, and accuracy values were computed and achieved as 0.6687, 0.9835, and 0.95570, respectively, for DRIVE database. Similarly, Table 2 shows the performance measures of the original curvelet transform on CHASE_DB1 database. The average sensitivity, specificity, and accuracy values were computed and found to be 0.6160, 0.9694, and 0.9432, respectively, for CHASE_DB1 database.

The second experiment is conducted for the proposed curvelet transform integrated with the Jerman filter. Table 3 shows the results of the average sensitivity, specificity, and accuracy values that are boosted to 0.7528, 0.9933, and 0.96008, respectively on DRIVE database. Likewise, Table 3 shows the results of average sensitivity, specificity, and accuracy values are boosted to 0.7078, 0.9850, and 0.9559, respectively on CHASE_DB1 database. For the calculation of the average values of the performance parameters, the highest values achieved from the various values of τ are considered. All the performance parameters are calculated with τ value between 0.5 and 1. The cut-off threshold value τ can be varied from zero to one. However, experimentally, it is observed that selecting a low value of τ below 0.5 supresses the elongated structures, and a high value supresses the bifurcation response. It is observed that better segmentation is achieved when the τ value is on the lower side, i.e., 0.5; poor segmentations are achieved as the τ value is increased and on the higher side. As the image illuminations vary for different images, various values of τ are utilized in the experiment. Accordingly, the image can be enhanced, and the value of τ can be fixed. The enhancement function of the multiscale filter (Function (1)) is calculated with scales from s_min_ = 3 to s_max_ = 16 pixels with step 0.5. 

## 4. Discussions

**Figure 7:**
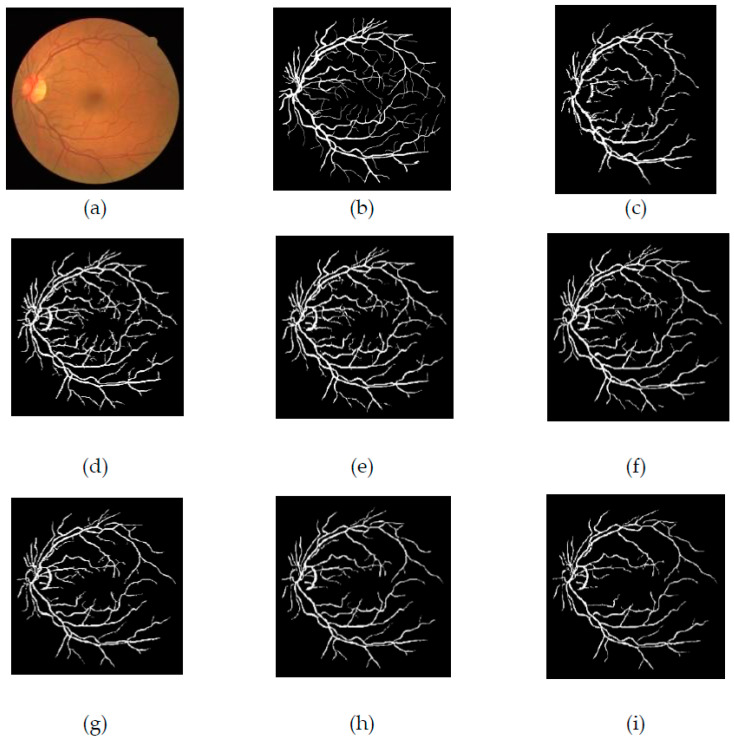
(**a**) Original image of DRIVE dataset, (**b**) Ground truth image, (**c**) Segmented image from original curvelet, (**d**) Segmented image of the suggested technique with τ = 0.5, (**e**) Segmented image of the suggested technique with τ = 0.6, (**f**) Segmented image of the suggested technique with τ = 0.7, (**g**) Segmented image of the suggested technique with τ = 0.8, (**h**) Segmented image of the suggested technique with τ = 0.9, (**i**) Segmented image of the suggested technique with τ = 1.

**Figure 8:**
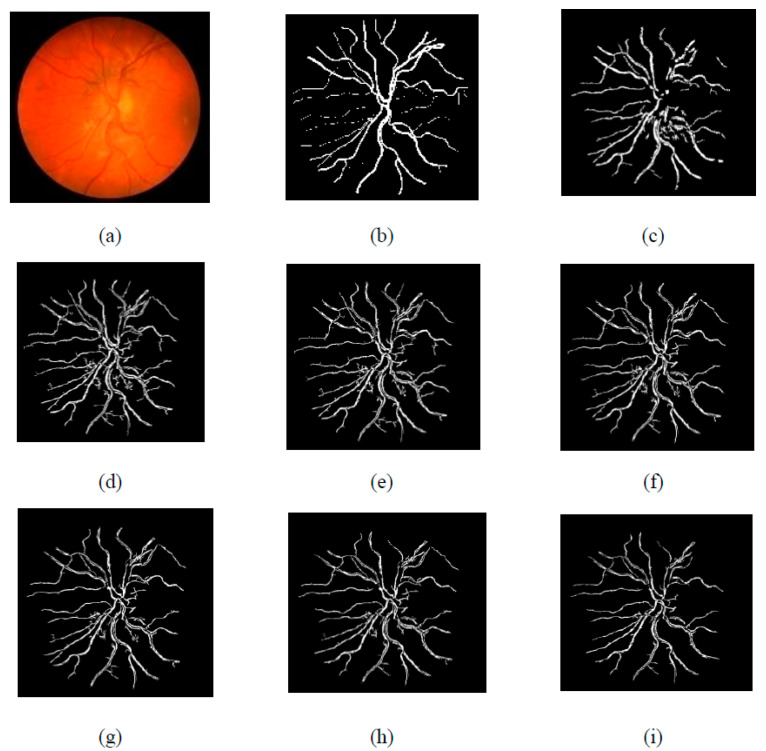
(**a**) Original image of CHASE-DB1 dataset, (**b**) Ground truth image, (**c**) Segmented image from original curvelet, (**d**) Segmented image of the suggested technique with τ = 0.5, (**e**) Segmented image of the suggested technique with τ = 0.6, (**f**) Segmented image of the suggested technique with τ = 0.7, (**g**) Segmented image of the suggested technique with τ = 0.8, (**h**) Segmented image of the suggested technique with τ = 0.9, (**i**) Segmented image of the suggested technique with τ = 1.

The segmented images of the DRIVE and CHASE_DB1 databases by the suggested approach are represented in Figure 7 and Figure 8, respectively. The first retinal original image of the DRIVE database is given in Figure 7a, while Figure 7b is the corresponding ground truth image. Figure 7c represents the vessel extracted by applying the curvelet transform enhanced method. Figure 7d–i shows the vessels extracted by the suggested method with different τ values of the Jerman filter. From the figures, it is clearly visible that, when increasing the τ values from 0.5 to 1 with an increment step of 0.1, the small branching vessels are disconnected from the main vessels, and correspondingly, the accuracy also reduced. The τ value at 0.5 finely preserved the vessel connectivity that is noticeably observed from Figure 7d. Furthermore, when the vessel-extracted image 7d is compared with the ground truth image 7b, it is noted that the thin vessels are more prominently detectable in Figure 7d.

For Figure 7, the first retinal original image of the CHASE_DB1 database is given in Figure 7a, while Figure 7b is the corresponding ground truth image. Figure 7c represents the vessel extracted by applying curvelet transform enhanced method. Figure 7d–i shows the vessels extracted by the suggested method with different τ values of the Jerman filter. All the observations of Figure 8 for CHASE_DB1 database are as explained for DRIVE database.

Thus, experimentally, it is noted that the existing curvelet transform enhancement method fail to extract many thin vessels accurately. The main advantage of the suggested approach is that it does not have postprocessing module after segmentation.

Table 3 represents the comparison of the performance measures of the recommended approach (that is computed for 0.5 as τ value) with other approaches presented in the literature. The performance of the proposed technique on DRIVE and CHASE_DB1 datasets are related with other techniques with reference to sensitivity, specificity, and accuracy: Kar et al. [12], Azzopardi et al. [40], Mapayi et al. [41], Zhao et al. [42], Zhang et al. [43], Tan et al. [44], Farokhain et al. [45], Orlando et al. [46], Rodrigues and Marengoni [47], Jiang et al. [48], Khomri et al. [49], Memari et al. [50], Sundaram et al. [51], Dash and Senapati [52], and Dash et al. [53]. It establishes that the recommended method accomplishes a higher accuracy in comparison with many state-of-the-art methods while retaining comparable sensitivity and specificity value.

As the suggested work is to improve the traditional curvelet transform, from Table 3 it is observed that there is a significant improvement in the results by the suggested method compared to traditional curvelet transform technique both for the DRIVE and CHASE_DB1 databases. Furthermore, the suggested approach outperforms the state-of-the-art-of methods.

## 5. Conclusions

This paper recommends a new technique for making curvelet transform approach more robust for retinal blood vessel segmentation by integrating it with the Jerman filter for different cut-off threshold values (τ) of the Jerman filter. For segmentation purpose, mean-C thresholding technique is employed. The integration of the Jerman filter and curvelet decomposition strongly intensify both thick and thin vessels and hence delivers better segmentation performance than the original curvelet transform. Simulation results establish that the suggested integrated scheme effectively detects the blood vessels and outperforms the state-of-the-art approaches in terms three performance indicators, sensitivity, accuracy, and specificity, over two public databases, DRIVE and CHASE_DB1. The achieved average sensitivity, specificity, and accuracy of segmented images are 0.7528, 0.9933, 0.9600 and 0.7078, 0.9850, 0.9559 on DRIVE and CHASE_DB1 databases, respectively. 

Additionally, the proposed approach can be employed in a real-time scenario, as the approach is an unsupervised technique that does not need any training data.

Even though the segmentation performances are promising, they can further be improved by considering the optimum set of curvelet coefficients. In the future, this approach can be extended for classification of abnormal and healthy image by assimilating deep-learning-based classifiers.

## Figures and Tables

**Table 1:** Performance evaluation of the original curvelet transform on DRIVE database.

Image	Sensitivity	Specificity	Accuracy	Precision
Retina 1	0.689029	0.983848	0.957543	0.806914
Retina 2	0.681326	0.98826	0.956828	0.868788
Retina 3	0.662299	0.982472	0.950555	0.807091
Retina 4	0.612176	0.994229	0.959083	0.914874
Retina 5	0.625776	0.99155	0.957283	0.884459
Retina 6	0.619286	0.98656	0.950812	0.832454
Retina 7	0.645363	0.982846	0.952006	0.790952
Retina 8	0.646095	0.981693	0.952819	0.768638
Retina 9	0.64762	0.985057	0.95771	0.792622
Retina 10	0.619679	0.989112	0.958707	0.836174
Retina 11	0.663699	0.98082	0.952431	0.772854
Retina 12	0.690874	0.979726	0.954785	0.763055
Retina 13	0.560464	0.990786	0.948715	0.868271
Retina 14	0.751659	0.969751	0.952118	0.686101
Retina 15	0.714534	0.973168	0.954658	0.672419
Retina 16	0.680373	0.984199	0.956767	0.810371
Retina 17	0.665984	0.977015	0.950761	0.727612
Retina 18	0.703259	0.97927	0.957401	0.744855
Retina 19	0.804903	0.986169	0.971133	0.840365
Retina 20	0.69116	0.983526	0.962026	0.769065

**Table 2:** Performance evaluation of the original curvelet transform on CHASE_DB1 database.

Image	Sensitivity	Specificity	Accuracy	Precision
Retina 1	0.605098	0.969179	0.943788	0.595448
Retina 2	0.588396	0.957322	0.923329	0.526972
Retina 3	0.670129	0.969808	0.946311	0.653783
Retina 4	0.622163	0.978558	0.947489	0.697793
Retina 5	0.632404	0.971317	0.943688	0.651083
Retina 6	0.582326	0.978046	0.944288	0.66521
Retina 7	0.609928	0.968976	0.94101	0.624149
Retina 8	0.613154	0.972105	0.947597	0.595198
Retina 9	0.600965	0.970603	0.951395	0.567225
Retina 10	0.597453	0.963862	0.937151	0.588464
Retina 11	0.619103	0.961833	0.940899	0.556381
Retina 12	0.592585	0.965196	0.937655	0.564901
Retina 13	0.597962	0.973663	0.947481	0.576893
Retina 14	0.692803	0.972191	0.95296	0.648084

**Table 3:** Performance Comparison of the recommended approach.

Approach	Year	Sensitivity		Specificity		Accuracy	
		DRIVE	CHASE_ DB1	DRIVE	CHASE_DB1	DRIVE	CHASE_DB1
Kar et al. [12]	2016	0.7548	--	0.9792	--	0.9616	--
Azzopardi et al. [40]	2015	0.7655	0.7585	0.9704	0.9587	0.9442	0.9387
Mapayi et al. [41]	2015	0.7650	--	0.9724	--	0.9511	--
Zhao et al. [42]	2015	0.742	--	0.982	--	0.954	--
Zhang et al. [43]	2016	0.7473 0.7743	0.7626 0.7277	0.9764 0.9725	0.9661 0.9712	0.9474 0.9476	0.945 --
Tan et al. [44]	2016	0.7743	0.7626	0.9725	0.9661	0.9476	0.9452
Farokhain et al. [45]	2017	0.693	--	0.979	--	0.939	--
Orlando et al. [46]	2017	0.7897	0.7277	0.9684	0.9712	--	--
Rodrigues and Marengoni [47]	2017	0.7223	--	0.9636	--	0.9472	--
Jiang et al. [48]	2018	0.7121	0.7217	0.9832	0.9770	0.9593	0.9591
Khomri et al. [49]	2018	0.739	--	0.974	--	0.945	--
Memari et al. [50]	2019	0.761	0.738	0.981	0.968	0.961	0.939
Sundaram et al. [51]	2019	0.69	0.71	0.94	0.96	0.93	0.95
Dash and Senapati [52]	2020	0.7403	--	0.9905	--	0.9661	--
Dash et al. [53]	2020	0.7203	0.6454	0.9871	0.9799	0.9581	0.9609
Original Curvelet Transform		0.6687	0.6160	0.9835	0.9647	0.9557	0.9432
Suggested approach (Jerman filter integrated with Curvelet transform)		0.7528	0.7078	0.9933	0.9850	0.9600	0.9559

## Data Availability

Publicly available datasets were analysed in this study.
